# Video slot machine use in adolescence: the role of self-efficacy beliefs, current and expected personal fulfillment at the social and educational level

**DOI:** 10.1016/j.abrep.2024.100560

**Published:** 2024-07-16

**Authors:** Giansanto Mosconi, Joseph DelFerro, Andrea Jin, Paola Bertuccio, Anna Odone, the Selfie Project Investigators, Ilaria Albertin, Andrea Amerio, Paola Bertuccio, Lorella Cecconami, Marcello Esposito, Simone Feder, Silvano Gallus, Sabrina Molinaro, Giansanto Mosconi, Anna Odone, Anna Polgatti, Sara Russo, Franco Taverna, Diego Turcinovich, Tomaso Vecchi

**Affiliations:** aa Semi di Melo Centre for Childhood and Adolescence Education and Research, Milan, Italy; ab Department of Neuroscience, Rehabilitation, Ophthalmology, Genetics, Maternal and Child Health (DINOGMI), Section of Psychiatry, University of Genoa, Genoa, Italy; ac IRCCS Ospedale Policlinico San Martino, Genoa, Italy; ad Department of Public Health, Experimental and Forensic Medicine, University of Pavia, Pavia, Italy; ae Health Protection Agency of Pavia, Pavia, Italy; af Department of Medical Epidemiology, Istituto di Ricerche Farmacologiche Mario Negri IRCCS, Milan, Italy; ag Institute of Clinical Physiology, National Research Council, Pisa, Italy; ah Medical Direction, Fondazione IRCCS Policlinico San Matteo, Pavia, Italy; ai Department of Brain and Behavioral Sciences, University of Pavia, Pavia, Italy; aj IRCCS Mondino Foundation, Pavia, Italy; a Department of Public Health, Experimental and Forensic Medicine, University of Pavia, Pavia, Italy; b Perelman School of Medicine, Department of Public Health, University of Pennsylvania, Philadelphia, USA; c Accademia Nazionale di Medicina, Genova, Italy; d Medical Direction, Fondazione IRCCS Policlinico San Matteo, Pavia, Italy

**Keywords:** Gambling, Slot machine, Adolescents, Cross-sectional study, Psychology, Associated factors

## Abstract

**Introduction:**

Video slot machines (VSMs), which are available on electronic gaming machines and online platforms, are widely recognized for their high potential to trigger harmful gambling behaviors. Yet, the psychological factors associated with VSM use among adolescents remain poorly investigated.

**Methods:**

We estimated adjusted odds ratios (OR) and corresponding 95% confidence intervals (CI) of current regular (at least monthly) VSM use according to self-efficacy beliefs regarding everyday problem-solving ability, along with current and expected personal fulfillment at the social, and educational level among 7,959 Italian high school students aged 15–17.

**Results:**

Current regular VSM use was reported by 108 students (1.4%). Notably, this behavior was more frequently reported by those with either particularly high or low self-ratings in problem-solving ability, current and expected social fulfillment, as well as by those with lower self-ratings in both current and expected educational/work fulfillment.

**Conclusions:**

These findings underscore that during adolescence, gambling behaviors are associated with diverse psychological profiles, each entailing distinct vulnerabilities. Effectively addressing adolescent gambling necessitates a comprehensive approach, with a strong emphasis on educational support. Given the increasing challenges in curbing underage exposure and access to the most harmful gambling formats, this study offers valuable insights for the design of health promotion and prevention strategies.

## Highlights

• Video Slot Machines (VSM) might be accessed by many Italian underage students.
• Self-efficacy beliefs on problem-solving ability vary among VSM users.
• VSM use relates variably to current and expected social fulfillment.
• VSM use is linked to lower current and expected educational fulfillment.

## 1 Introduction

Despite its illegality, underage gambling is highly prevalent across Italy: in 2022, around 50 % of students aged 15–17 reported gambling within the past 12 months (ESPAD, 2023). Poor enforcement of age restrictions, readily available online gambling platforms, and public acceptance facilitate early establishment of dangerous gambling patterns, leading a considerable share of youths to gamble monthly (Canale et al., 2017, Delfabbro et al., 2016).

In Italy, Video slot machines (VSM), which are presumed to have high addictive power due to high event frequency, cheap buy-in, and short intervals between stake and payout (Murch and Clark, 2021, Yücel et al., 2018), are available on Amusement With Prizes (AWPs) machines, Video Lottery Terminals (VLT) or online gambling platforms. In an effort to curb underage access to AWPs and VLTs, specific local regulations and national policies have been recently implemented (Tavazzani et al., 2020); however, the effectiveness of such measures is a subject of disagreement (Marionneau et al., 2022, Rolando et al., 2020). Increasingly popular online platforms also present new challenges for enforcing restrictions (Delfabbro et al., 2016). Hence, it is essential to gain a comprehensive understanding of factors influencing adolescents' VSM use to develop effective educational strategies for discouraging participation in this harmful gambling format, particularly in cases where legal measures fall short.

Prior research on adolescent gambling has largely investigated its relationships with demographic factors, overt psychiatric disorders, and specific personality traits such as impulsivity (Ferrara et al., 2018, Riley et al., 2021). However, these variables may not fully capture an adolescent’s global well-being, which is influenced by many cognitive and dispositional factors (Donati et al., 2018, Proctor et al., 2008).

Within social-cognitive theory, self-efficacy reflects an individual's confidence in their ability to achieve desired outcomes (Bandura, 1977). Research suggests that general self-efficacy, which is linked to problem-solving ability and represents global confidence in one's coping capacity across a wide range of demanding, unexpected, and novel situations, is a key predictor of positive adjustment during adolescence and successful outcomes across various life domains, even in unfavorable circumstances (Marcionetti and Rossier, 2019, Pajeres and Urdan, 2006). Studies on gambling have mainly focused on self-efficacy in relation to gamblers' confidence in their ability to regulate their gambling behavior and avoid gambling in risky situations (Barbaranelli et al., 2017, Casey et al., 2007, Parrado-González et al., 2023). Alternatively, some authors have focused on social and academic self-efficacy, which respectively reflect confidence in one's ability to have satisfactory relationships and perform well in the academic context, revealing an inverse relationship with problem gambling during adolescence (Bozzato et al., 2020, Passanisi et al., 2020). However, to the best of our knowledge, there is currently little discussion on the role of general self-efficacy in gambling behaviors (Huic et al., 2017), particularly regarding how adolescents' gambling behaviors may be influenced by their self-efficacy beliefs concerning everyday problem-solving. Exploring this connection could provide a more comprehensive insight into their resilience across various life domains (Sagone et al., 2020).

Amid the factors commonly recognized to play a central role in adolescents' well-being, enriching academic experience and satisfying social connections stand out prominently (Allen et al., 2024, Avedissian and Alayan, 2021, Webster and Dunne, l., & Hunter, R. , 2020). To date, research on gambling has largely overlooked adolescents' personal fulfillment in these domains, primarily relying on externally defined measures of social functioning and academic achievement (Riley et al., 2021). However, such an approach neglects the subjective component of wellbeing (Das et al., 2020). In fact, objective measures often represent distal outcomes influenced by a complex interplay of factors beyond individual control. By contrast, self-reported assessments of well-being, such as those evaluating personal fulfillment, may provide a closer and more nuanced insight into subjective experiences, potentially uncovering unmet needs (Das et al., 2020, Ortuño-Sierra et al., 2020).

Addressing the gaps in adolescent gambling literature, the current exploratory study aims to investigate how (1) self-rated everyday problem-solving capacity, (2) current and (3) expected personal fulfillment across social, and school/work domains related to regular gambling (i.e. monthly or more frequent) with VSMs in a sample of almost 8,000 Italian high school students aged 15–17.

## 2 Methods

### 2.1 Study design, setting, and population

We conducted a repeated cross-sectional study using data collected from two surveys carried out in 2018 and 2022 in 11 high school in Pavia (Northern Italy) by the Semi di Melo Association as part of the “Selfie Project”. This initiative aims to gather data on the lifestyles, social interactions, and mental well-being of Italian middle and high school students through periodic surveys conducted in several Italian cities, with the goal of raising awareness of potential risks during adolescence. A comprehensive description of the features and objectives of the “Selfie Project” can be found elsewhere (Feder et al., 2023). Each school board approved participation in the “Selfie Project”. After merging data collected from the two surveys, the final sample comprised 7,959 students aged 15–17 years. There was no overlap between participants in the two surveys.

### 2.2 Procedure

Students were provided with a clear explanation of the research purpose and offered the opportunity to participate in the study by completing a fully anonymous online questionnaire accessible on school computers. Written informed consent was collected by all study participants. Participation in the study was on a voluntary basis, fully anonymous and contingent upon informed consent for data processing for research purposes through an electronic form. Data were collected during school hours. No compensation was provided for participating in the study. The project was approved by all included school boards and by the University and the Declaration of Helsinki was adequately addressed at all steps.

### 2.3 Measures

The questionnaire explored demographic characteristics, family context, educational background, and risky behaviors, including gambling. In the section on gambling, the questionnaire investigated the current frequency of use of different types of gambling games, including VSM.

The question on the frequency of VSM use within the last year was formulated to integrate widely recognized definitions of gambling formats within this category, aimed at improving understanding. The response options included were as follows: 1) “never,” 2) “less than once a month,” 3) “1 to 4 times a month,” 4) “2 to 4 times a week,” and 5) “every day or almost every day” The outcome in this study was “current regular VSM use”, defined as at least once per month use and obtained by grouping response options number 3, 4 and 5 from the previous question (then coded as a binary variable). We opted to focus on monthly VSM use to identify habitual users and distinguish them from those who gambled occasionally or only experimented with VSMs a few times in the past year. This definition of regular users aligns with established conventions in the field, as seen in studies by Chóliz et al. and Mazar et al. (Chóliz et al., 2022, Mazar et al., 2020), where monthly gambling frequency is used as the benchmark for regularity.

As potential associated factors, we considered self-rated everyday problem-solving ability, current and expected personal fulfillment at the social, and educational/work level. Participants rated their everyday problem-solving ability by responding to the question: “How would you rate your ability to deal with everyday problems?”. Current personal fulfillment was assessed with the following questions: “How would you rate your personal fulfillment at the social level?” and “How would you rate your personal fulfillment at the school level?”. Expected personal fulfillment was probed with the following questions: “What do you anticipate your fulfillment at the social level will be like in the future?” and “What do you anticipate your fulfillment in work or educational settings will be like in the future?”. The participants used a 4-point Likert scale (encompassing the options “high”, “fair”, “low”, “very low”) to answer the questions.

### 2.4 Statistical analysis

To estimate odds ratios (OR) and their corresponding 95 % confidence intervals (CI) for the associations between the investigated psychological factors and current regular VSM use, we carried out logistic regression models adjusted by survey year (2018 or 2022), sex, age, nationality, and type of school attended. The response “fair” was used as the reference category. To assess the presence of multicollinearity among the independent variables, we calculated the Variance Inflation Factor (VIF) for each covariate in the model and a collinearity diagnostic analysis based on eigenvalues and condition indices (Table S4 and Table S5). Finally, a power analysis was also performed. Given a sample size of 7,959 and that VSM regular use had a prevalence of 1.4 %, the study could detect effects with a margin of error of 0.26 % at a 95 % confidence level. All statistical analyses were performed using SAS 9.4 (Cary, NC, USA).

## 3 Results

Distribution of selected characteristics of the 7,959 high school students aged 15 to 17 is given in Table 1. About 44 % of the students were males, approximately half attended a lyceum, one-third attended a technical high school, and the remainder a vocational high school. Most students performed adequately in school, although 38.1 % had failed either a course or an entire year. Most households had two working parents and included at least one sibling. Table S1 presents the distribution of selected characteristics and the psychological variables investigated based on the survey year.

**Table 1:** Distribution of 7,959 high school students aged 15–17, by selected demographic, family, and educational characteristics. Pavia, Lombardy region, Italy (2018–2022).

	**n**	**%**
**Overall**	7,959	100
**Sex**		
Male	3,516	44.2
Female	4,443	55.8
**Nationality**		
Italian	6,868	86.3
Foreign (born in Italy)	640	8.0
Foreign (born abroad)	451	5.7
**Type of school attended**		
Lyceum	3,781	47.5
Technical college	2,466	31.0
Vocational college	1,712	21.5
**School performance**		
Never failed a year or a course	4,927	61.9
Never failed a year but failed a course	2,050	25.8
Failed a year	982	12.3
**Parental employment**		
Both parents working	5,056	63.5
One parent working	2,138	26.9
Other	765	9.6
**Household composition**		
Both parents plus (a) sibling(s)	4,757	59.8
Both parents and no siblings	1,668	21.0
One parent plus (a) sibling(s)	934	11.7
One parent and no siblings	600	7.5

**Table 2:** Overall distribution (n) of 7,959 high school students aged 15–17 according to self-rated problem-solving capacity, present and expected personal fulfillment, and prevalence (Prev.) of current regular VSM^#^ use, along with adjusted* odds ratios (OR) and corresponding 95% confidence intervals (CI). Pavia, Lombardy region, Italy (2018; 2022).

	Current regular VSM use				
	n	Prev.	OR* (95 % CI)	*p*-value	AUC^
**Total**	7,959	1.4			
					
**Self-rated problem-solving ability**					0.764
High	2,015	2.1	**1.77 (1.17 – 2.69)**	**0.007**	
Fair	4,761	1.1	1.00°	−	
Low	925	0.9	0.89 (0.42 – 1.89)	0.758	
Very low	258	3.1	**3.00 (1.40 – 6.47)**	**0.005**	
					
**Present personal fulfillment at the social level**					0.770
High	1,747	2.2	**2.36 (1.47 – 3.77)**	**<0.001**	
Fair	4,022	0.8	1.00°	−	
Low	1,540	1.4	**1.97 (1.14 – 3.41)**	**0.015**	
Very low	650	2.2	**2.85 (1.50 – 5.38)**	**0.001**	
					
**Present personal fulfillment at the educational level**					0.763
High	1,158	1.3	1.26 (0.68 – 2.30)	0.464	
Fair	3,775	1.0	1.00°	−	
Low	1,971	1.7	**1.67 (1.04 – 2.69)**	**0.034**	
Very low	1,054	2.2	**2.10 (1.24 – 3.57)**	**0.006**	
					
**Expected future personal fulfillment at the social level**					0.779
High	3,417	1.5	**1.93 (1.21 – 3.09)**	**0.006**	
Fair	3,529	0.8	1.00°	−	
Low	714	2.4	**3.23 (1.74 – 5.99)**	**<0.001**	
Very low	299	4.0	**4.69 (2.33 – 9.44)**	**<0.001**	
					
**Expected future personal fulfillment at the educational/work level**					0.783
High	3,974	1.2	1.51 (0.93 – 2.46)	0.096	
Fair	3,023	0.9	1.00°	−	
Low	630	2.9	**3.07 (1.67 – 5.66)**	**<0.001**	
Very low	332	5.4	**5.47 (2.94 – 10.16)**	**<0.001**	

^#^ Video slot machines include: Amusement With Prize (AWPs) machines, Video Lottery Terminals (VLTs) and online slot machines.

* Estimated through logistic regression models adjusted by year of interview (2018, 2022), sex (male, female), age (continuous), nationality (Italian, foreign born in Italy, foreign born abroad), and type of school attended (lyceum, technical college, vocational college). Estimates in bold are statistically significant at 0.05 level.

° Reference category.

^Area under the curve of each model.

Out of the total participants, 7,502 students (94.3 %) reported never using VSMs, while 349 (4.4 %) reported using them less than monthly. Out of the 108 (1.4 %) students reporting VSM use at least once a month (i.e., current regular VSM use), 59 stated they used VSMs from 1 to 4 times per month, 25 reported using them 5 to 7 times a month, and 24 reported using them every day or almost every day. Table S2 presents the distribution of selected characteristics and psychological variables among subjects reporting current regular VSM use compared to those not reporting it. Table 2 reports the percentage of current regular VSM use and the adjusted odds ratios (OR) with their 95 % confidence intervals (CI) for associations with self-rated everyday problem-solving ability and both present and expected personal fulfillment at the social and the educational/work level.

Regarding self-rated everyday problem-solving ability, as compared to “fair” ratings, both “high” and “very low” ratings were more frequently associated with regular VSM use (OR: 1.77, 95 % CI: 1.17–2.69 and OR: 3.00, 95 % CI: 1.40–6.47, respectively). Similarly, compared to students who rated their current personal fulfillment at the social level as “fair”, those who rated it as “high”, “low”, and “very low” were more frequently current regular VSM users (OR: 2.36, 95 % CI: 1.47–3.77; OR: 1.97, 95 % CI: 1.14–3.41; OR: 2.85, 95 % CI: 1.50–5.38, respectively). At the educational level, compared to students who rated their current personal fulfillment as “fair”, those who rated it as “low” and “very low” were more frequently current regular VSM users (OR: 1.67, 95 % CI: 1.04–2.69 and OR: 2.10, 95 % CI: 1.24–3.57, respectively). Concerning future social fulfillment, students who expected it to be “high”, “low”, and “very low” were more frequently current regular VSM users (OR 1.93, 95 % CI 1.21–3.09; OR 3.23, 95 % CI 1.74–5.99; and OR 4.69, 95 % CI 2.33–9.44, respectively) than those who expected it to be “fair”. Regarding future educational/work fulfillment, both “low” and “very low” expectations were also more frequently associated with current regular VSM use, compared to “fair” expectations (OR 3.07, 95 % CI 1.67–5.66 and OR 5.47, 95 % CI 2.94–10.16, respectively).

Table S3 shows the distribution of current and expected personal fulfillment in education and work according to the type of high school attended. There are significant differences, with students from vocational high schools reporting lower current fulfillment than students in lyceums and lower expected fulfillment than students in lyceums or technical high schools.

## 4 Discussion

In our large sample of adolescents, regular VSM use was more frequently reported among individuals with the highest and lowest self-ratings in problem-solving ability, as well as those with the highest and lowest levels of current and expected social fulfillment. When considering the educational/work dimension, we found that regular VSM use was more frequent among participants reporting lower levels of both current and expected fulfillment.

While the literature extensively documents the role of higher levels of self-efficacy for gambling engagement and gambling regulation in mitigating gambling's detrimental effects and predicting the success of treatment programs (de Ridder & Deighton, 2022), the role of general self-efficacy within this context remains under-explored. The finding that lower self-ratings in everyday problem-solving ability were linked to regular VSM use appears to align with evidence supporting the significance of strong general self-efficacy in protecting against risky behaviors during adolescence (Pajeres & Urdan, 2006). Nonetheless, regular VSM use was also more frequent among those reporting high self-rated everyday problem-solving ability. This might be explained by the fact that some gamblers are prone to experience cognitive distortions which lead them to perceive themselves as having higher cognitive abilities (Cosenza and Nigro, 2015, Passanisi et al., 2020).

Regarding the social domain, the results were also mixed. The link between a low or very low self-rating in both current and expected social fulfillment might be elucidated by the fact that adolescents' perceived social self-efficacy (their confidence in their ability to conduct satisfactory social interactions) decreases with engagement in gambling, and low social self-efficacy might influence maladaptive coping strategies that foster addictive behaviors (Passanisi et al., 2020). Nevertheless, our results also indicated a strong association between high perceived social fulfillment and regular VSM use. Indeed, gambling can be supported by peer groups, and perceived peer approval of gambling is associated with increased gambling frequency (Khasmohammadi et al., 2020, Marinaci et al., 2021). Adolescent social dynamics may have either positive or negative influences on gambling behavior, depending on social context and norms (Emond & Griffiths, 2020).

Clarifying the nature of the direct association between current low educational fulfillment and VSM use is challenging. On the one hand, there exists the potential for a shared psychological foundation. This connection may be attributed to factors such as impulsivity, identified by certain authors as a compelling driver for participation in gambling activities (Emond & Griffiths, 2020), and concurrently associated with diminished interest in school activities, school misconduct, and suboptimal educational achievement (Marriott et al., 2019, Vogel and Barton, 2013). On the other hand, involvement in gambling activities can divert adolescents from their academic responsibilities, detrimentally impacting their school performance (Vitaro et al., 2018). However, assuming that the association could also play out in the opposite direction, it is possible that a caring, stimulating, and inclusive school environment could act as a protective factor, discouraging adolescents from engaging in this risky behavior. Indeed, research indicates that high levels of teacher support and teacher caring can protect youth from engaging in gambling (Elgar et al., 2018, Räsänen et al., 2016, Wahlström et al., 2022, Wahlström and Olsson, 2023). Our findings on the connection between school dissatisfaction and regular VSM use are further supported by additional data (as shown in Table S2). This data indicates a link between past academic struggles (such as failing a course or a year) and regular VSM use, consistent with previous studies (De Luigi et al., 2018, Livazović and Bojčić, 2019).

In regard to expected fulfillment in both social and educational/work domains, low and very low self-ratings were consistently associated with regular VSM use, in line with a similar finding that positive future orientation was inversely associated with adolescent gambling frequency (Donati et al., 2018). It is possible that negative future views lead to the feeling of having “nothing to lose” and engagement in activities that provide immediate gratification (Brolin Låftman et al., 2020). However, compared to those rating their expected social fulfillment as “fair”, participants perceiving it as “high” more frequently reported regular VSM use. One study similarly reported that gambling was more prevalent among those with the most optimistic future orientation, but the association did not hold for at-risk gambling, a distinction which was not measured in the present study (Brolin Låftman et al., 2020). Perhaps the association with expected social fulfillment varies between sub-types, as non-problem gamblers may gamble more socially than often-isolated problem gamblers.

The multifaceted relationship that has emerged between self-perception of everyday problem-solving ability, both current and expected personal fulfillment at the social level and VSM use, supports the concept that adolescent gambling behaviors may be linked to diverse psychological profiles, each accompanied by unique needs and vulnerabilities. In 2002, Blaszczynski and Nower introduced the Pathways Model, which focused on how biological, psychological, and environmental factors may combine in different ways to influence the development of gambling tendencies (Blaszczynski & Nower, 2002). Building upon this theoretical framework, findings from recent studies support the existence of distinct profiles among gamblers, each exhibiting unique behavioral characteristics (Aonso-Diego et al., 2024, Devos et al., 2020). This evidence suggest heterogeneity in gambling motivations, with some individuals seeking entertainment and others potentially using gambling to manage stress, anxiety, or depression. In this regard, it is worth noting that subjects participating in the second survey have been exposed to the COVID-19 pandemic and subsequent social distancing measures, as well as disruptions to education and social activities. These factors might have exacerbated pre-existing vulnerabilities, pushing some individuals towards online VSMs as a coping strategy. When interpreting our results, it must also be considered that adolescents who gamble may exhibit varying susceptibility to cognitive distortions, leading to both optimistic and pessimistic perceptions about themselves (Mallorquí-Bagué et al., 2019, Mathieu et al., 2020). This may be particularly evident in domains like self-efficacy and social fulfillment, which rely heavily on self-evaluation compared to external judgments such as grades in school. Moreover, recent research suggests that individuals with severe gambling problems may experience dysregulation of positive emotions. These individuals often negatively judge their positive emotional states and resort to gambling as a means to avoid the arousal associated with positive emotions (Rogier et al., 2022), and this might have potentially impacted our results.

Perceptions of fulfillment in the school/work domain appear to play a more specific role, as only lower self-ratings in this area showed a significant direct association with VSM use. These findings suggest that addressing adolescent gambling requires particular attention to educational factors and that directing preventive efforts towards the school environment could prove highly beneficial. Unfortunately, although gambling poses numerous risks with potential long-term health consequences, teachers generally do not perceive it as a significant concern when compared to other risky behaviors (Campbell et al., 2011, Derevensky et al., 2014, Räsänen et al., 2015). Fostering awareness on this topic within the school environment is paramount, since teachers can wield influence over student behavior, and peer pressure can contribute to the adoption of risky behaviors (Emond and Griffiths, 2020, Roberts et al., 2023, Tani et al., 2021).

The existing literature is limited by its use of external assessments of fulfillment and well-being. Our study overcomes this by assessing self-perception in psychological domains important to adolescent development. Another strength is the focus on highly addictive VSMs, as previous studies have measured gambling more generally. However, this study is not without limitations. First, the use of cross-sectional data from a convenience sample of only students in Pavia prevents the determination of causal relationships and limits generalizability. The temporality of the described associations is unknown and there exists potential for reverse or bidirectional influences. Second, psychological determinants were not assessed using validated scales, although survey questions were designed to connect to constructs previously shown to be important for adolescent well-being. Third, this study relies on self-report data, which is limited by social desirability bias, recall bias, and misinterpretation of questions. Attempts were made to limit these biases through emphasized anonymity.

Given that 8.7 % of adolescents aged between 15 and 19 reported having used VSM at least once during 2022 in the European School Survey Project on Alcohol and Other Drugs (ESPAD) Italy survey, this highly addictive gambling format remains largely accessible to adolescents in our country (ESPAD, 2023). Our findings can contribute to the design of effective preventive interventions aimed at safeguarding adolescents from the potential development of compulsive gambling behaviors stemming from regular VSM use. To gain deeper insights, future research should employ longitudinal studies to track gambling patterns over time. This would allow for a clearer understanding of how these patterns evolve, and pinpoint the specific timing of the relationships we identified in this study. Examining the differences in VSM use between land-based and online settings could provide deeper insights into the factors influencing this behavior.

## 5 Conclusions

To effectively address the problem of adolescent gambling in its most harmful forms, interventions must be personalized, sensitive to individual vulnerabilities, and heavily focused on educational components. Findings from the present study offer insights for a more profound comprehension of potential risk and protective factors, thereby assisting the design of health promotion and prevention initiatives for the implementation of which schools could provide an ideal environment (Pulimeno et al., 2020).

## The Selfie Project Investigators (corporate authorship)

Ilaria Albertin, Semi di Melo Centre for Childhood and Adolescence Education and Research, Milan, Italy; Andrea Amerio, Department of Neuroscience, Rehabilitation, Ophthalmology, Genetics, Maternal and Child Health (DINOGMI), Section of Psychiatry, University of Genoa, Genoa, Italy, IRCCS Ospedale Policlinico San Martino, Genoa, Italy; Paola Bertuccio, Department of Public Health, Experimental and Forensic Medicine, University of Pavia, Pavia, Italy; Lorella Cecconami, Health Protection Agency of Pavia, Pavia, Italy; Marcello Esposito, Semi di Melo Centre for Childhood and Adolescence Education and Research, Milan, Italy; Simone Feder, Semi di Melo Centre for Childhood and Adolescence Education and Research, Milan, Italy; Silvano Gallus, Department of Medical Epidemiology, Istituto di Ricerche Farmacologiche Mario Negri IRCCS, Milan, Italy; Sabrina Molinaro, Institute of Clinical Physiology, National Research Council, Pisa, Italy; Giansanto Mosconi, Department of Public Health, Experimental and Forensic Medicine, University of Pavia, Pavia, Italy; Anna Odone, Department of Public Health, Experimental and Forensic Medicine, University of Pavia, Pavia, Italy, Medical Direction, Fondazione IRCCS Policlinico San Matteo, Pavia, Italy; Anna Polgatti, Semi di Melo Centre for Childhood and Adolescence Education and Research, Milan, Italy; Sara Russo, Department of Public Health, Experimental and Forensic Medicine, University of Pavia, Pavia, Italy; Franco Taverna, Semi di Melo Centre for Childhood and Adolescence Education and Research, Milan, Italy; Diego Turcinovich, Semi di Melo Centre for Childhood and Adolescence Education and Research, Milan, Italy; Tomaso Vecchi, Department of Brain and Behavioral Sciences, University of Pavia, Pavia, Italy, IRCCS Mondino Foundation, Pavia, Italy.

## Funding

No funding was received to assist with the preparation of this manuscript.

## Ethics declarations

Written informed consent was collected by all study participants, this aligning to both Italian law and the General Data Protection Regulation (GDPR) of the European Union. Participation in the study was on a voluntary basis, fully anonymous (making it impossible to trace the identity of participants), and contingent upon informed consent for data processing for research purposes through an electronic form. The project was approved by all included school boards and by the University and the Declaration of Helsinki was adequately addressed at all steps.

## Data availability statement

The data that support the findings of this study are available from the corresponding author on reasonable request.

## Author agreement statement

We the undersigned declare that this manuscript is original, has not been published before and is not currently being considered for publication elsewhere.

We confirm that the manuscript has been read and approved by all named authors and that there are no other persons who satisfied the criteria for authorship but are not listed. We further confirm that the order of authors listed in the manuscript has been approved by all of us.

We understand that the Corresponding Author is the sole contact for the Editorial process.

He/she is responsible for communicating with the other authors about progress, submissions of revisions and final approval of proofs.

## CRediT authorship contribution statement

**Giansanto Mosconi:** Writing – original draft, Methodology, Data curation, Conceptualization. **Joseph DelFerro:** Writing – original draft. **Andrea Jin:** Writing – original draft. **Paola Bertuccio:** Writing – original draft, Methodology, Formal analysis, Data curation, Conceptualization. **Anna Odone:** Writing – review & editing, Supervision, Conceptualization. All the Selfie Project Investigators actively contributed to conducting the study, participating in data collection and interpretation, and reviewing the final draft.

## Declaration of competing interest

The authors declare that they have no known competing financial interests or personal relationships that could have appeared to influence the work reported in this paper.

## Data Availability

Data will be made available on request.
