# Supplementary material for: Video slot machine use in adolescence: the role of self-efficacy beliefs, current and expected personal fulfillment at the social and educational level
Source: Addict Behav Rep. 2024 Jul 16;20:100560. doi: 10.1016/j.abrep.2024.100560 (PMC11437869; doi:10.1016/j.abrep.2024.100560)
Supplement: Supplementary Data 1 [file mmc1.docx]

**SUPPLEMENTARY MATERIALS**

**Supplementary Table 1.** Distribution of selected characteristics and investigated psychological variables among 7,959 high school students aged 15-17, by survey year. Pavia, Lombardy region, Italy (2018-2022).

|  | **Survey year** | | | |  |
| --- | --- | --- | --- | --- | --- |
|  | **2018** | | **2022** | |  |
|  | **n** | **%** | **n** | **%** | ***p*-value (Chi-square test)** |
|  |  |  |  |  |  |
| **Sex** |  |  |  |  | **0.0020** |
| Male | 1422 | 42.2 | 2094 | 45.7 |  |
| Female | 1950 | 57.8 | 2493 | 54.3 |  |
|  |  |  |  |  |  |
| **Nationality** |  |  |  |  | **<.0001** |
| Italian | 2944 | 87.3 | 3924 | 85.5 |  |
| Foreign (born in Italy) | 197 | 5.8 | 443 | 9.7 |  |
| Foreign (born abroad) | 231 | 6.9 | 220 | 4.8 |  |
|  |  |  |  |  |  |
| **Type of school attended** |  |  |  |  | **0.0265** |
| Lyceum | 1562 | 46.3 | 2219 | 48.4 |  |
| Technical college | 1037 | 30.8 | 1429 | 31.2 |  |
| Vocational college | 773 | 22.9 | 939 | 20.5 |  |
|  |  |  |  |  |  |
| **School performance** |  |  |  |  | **<.0001** |
| Never failed a year or a course | 1990 | 59.0 | 2937 | 64.0 |  |
| Never failed a year but failed a course | 882 | 26.2 | 1168 | 25.5 |  |
| Failed a year | 500 | 14.8 | 482 | 10.5 |  |
|  |  |  |  |  |  |
| **Parental employment** |  |  |  |  | 0.0620 |
| Both parents working | 2097 | 62.2 | 2959 | 64.5 |  |
| One parent working | 927 | 27.5 | 1211 | 26.4 |  |
| Other | 348 | 10.3 | 417 | 9.1 |  |
|  |  |  |  |  |  |
| **Household composition** |  |  |  |  | 0.0596 |
| Both parents plus (a) sibling(s) | 2008 | 59.5 | 2749 | 59.9 |  |
| Both parents and no siblings | 673 | 20.0 | 995 | 21.7 |  |
| One parent plus (a) sibling(s) | 418 | 12.4 | 516 | 11.2 |  |
| One parent and no siblings | 273 | 8.1 | 327 | 7.1 |  |
|  |  |  |  |  |  |
| **Self-rated problem-solving ability** |  |  |  |  | 0.0647 |
| High | 820 | 24.3 | 1195 | 26.1 |  |
| Fair | 2075 | 61.5 | 2686 | 58.6 |  |
| Low | 372 | 11.0 | 553 | 12.1 |  |
| Very low | 105 | 3.1 | 153 | 3.3 |  |
|  |  |  |  |  |  |
|  |  |  |  |  |  |
| **Present personal fulfillment at the social level** |  |  |  |  | **<.0001** |
| High | 213 | 6.3 | 437 | 9.5 |  |
| Fair | 572 | 17.0 | 968 | 21.1 |  |
| Low | 1785 | 52.9 | 2237 | 48.8 |  |
| Very low | 802 | 23.8 | 945 | 20.6 |  |
|  |  |  |  |  |  |
| **Present personal fulfillment at the educational level** |  |  |  |  | **0.0250** |
| High | 431 | 12.8 | 623 | 13.6 |  |
| Fair | 791 | 23.5 | 1180 | 25.7 |  |
| Low | 1628 | 48.3 | 2147 | 46.8 |  |
| Very low | 522 | 15.5 | 636 | 13.9 |  |
|  |  |  |  |  |  |
| **Expected future personal fulfillment at the social level** |  |  |  |  | **<.0001** |
| High | 95 | 2.8 | 204 | 4.4 |  |
| Fair | 261 | 7.7 | 453 | 9.9 |  |
| Low | 1530 | 45.4 | 1999 | 43.6 |  |
| Very low | 1486 | 44.1 | 1931 | 42.1 |  |
|  |  |  |  |  |  |
| **Expected future personal fulfillment at the educational/work level** |  |  |  |  | 0.1532 |
| High | 124 | 3.7 | 208 | 4.5 |  |
| Fair | 253 | 7.5 | 377 | 8.2 |  |
| Low | 1298 | 38.5 | 1725 | 37.6 |  |
| Very low | 1697 | 50.3 | 2277 | 49.6 |  |
|  |  |  |  |  |  |

**Supplementary Table 2.** Distribution of selected characteristics and investigated psychological variables among 7,959 high school students aged 15-17 based on VSM use. Pavia, Lombardy region, Italy (2018-2022).

|  | **No VSM use or less than monthly VSM use** | | | **Current Regular VSM use** | | |  |
| --- | --- | --- | --- | --- | --- | --- | --- |
|  | **n** | **Column %** | **Row %** | **n** | **Column %** | **Row %** | ***p*-value (Chi-square test)** |
|  |  |  |  |  |  |  |  |
| **Sex** |  |  |  |  |  |  | **<.0001** |
| Male | 3428 | 43.7 | 97.5 | 88 | 81.5 | 2.5 |  |
| Female | 4423 | 56.3 | 99.5 | 20 | 18.5 | 0.5 |  |
|  |  |  |  |  |  |  |  |
| **Nationality** |  |  |  |  |  |  | 0.4132 |
| Italian | 6779 | 86.3 | 98.7 | 89 | 82.4 | 1.3 |  |
| Foreign (born in Italy) | 630 | 8.0 | 98.4 | 10 | 9.3 | 1.6 |  |
| Foreign (born abroad) | 442 | 5.6 | 98.0 | 9 | 8.3 | 2.0 |  |
|  |  |  |  |  |  |  |  |
| **Type of school attended** |  |  |  |  |  |  | **<.0001** |
| Lyceum | 3758 | 47.9 | 99.4 | 23 | 21.3 | 0.6 |  |
| Technical college | 2414 | 30.7 | 97.9 | 52 | 48.1 | 2.1 |  |
| Vocational college | 1679 | 21.4 | 98.1 | 33 | 30.6 | 1.9 |  |
|  |  |  |  |  |  |  |  |
| **School performance** |  |  |  |  |  |  | **<.0001** |
| Never failed a year or a course | 4887 | 62.2 | 99.2 | 40 | 37.0 | 0.8 |  |
| Never failed a year but failed a course | 2018 | 25.7 | 98.4 | 32 | 29.6 | 1.6 |  |
| Failed a year | 946 | 12.0 | 96.3 | 36 | 33.3 | 3.7 |  |
|  |  |  |  |  |  |  |  |
| **Parental employment** |  |  |  |  |  |  | **0.0110** |
| Both parents working | 4989 | 63.5 | 98.7 | 67 | 62.0 | 1.3 |  |
| One parent working | 2116 | 27.0 | 99.0 | 22 | 20.4 | 1.0 |  |
| Other | 746 | 9.5 | 97.5 | 19 | 17.6 | 2.5 |  |
|  |  |  |  |  |  |  |  |
| **Household composition** |  |  |  |  |  |  | 0.7748 |
| Both parents plus (a) sibling(s) | 4694 | 59.8 | 98.7 | 63 | 58.3 | 1.3 |  |
| Both parents and no siblings | 1646 | 21.0 | 98.7 | 22 | 20.4 | 1.3 |  |
| One parent plus (a) sibling(s) | 922 | 11.7 | 98.7 | 12 | 11.1 | 1.3 |  |
| One parent and no siblings | 589 | 7.5 | 98.2 | 11 | 10.2 | 1.8 |  |
|  |  |  |  |  |  |  |  |
| **Self-rated problem-solving ability** |  |  |  |  |  |  | **0.0003** |
| High | 1973 | 25.1 | 97.9 | 42 | 38.9 | 2.1 |  |
| Fair | 4711 | 60.0 | 98.9 | 50 | 46.3 | 1.1 |  |
| Low | 917 | 11.7 | 99.1 | 8 | 7.4 | 0.9 |  |
| Very low | 250 | 3.2 | 96.9 | 8 | 7.4 | 3.1 |  |
|  |  |  |  |  |  |  |  |
| **Present personal fulfillment at the social level** |  |  |  |  |  |  | **<.0001** |
| High | 1708 | 21.8 | 97.8 | 39 | 36.1 | 2.2 |  |
| Fair | 3989 | 50.8 | 99.2 | 33 | 30.6 | 0.8 |  |
| Low | 1518 | 19.3 | 98.6 | 22 | 20.4 | 1.4 |  |
| Very low | 636 | 8.1 | 97.8 | 14 | 13.0 | 2.2 |  |
|  |  |  |  |  |  |  |  |
| **Present personal fulfillment at the educational level** |  |  |  |  |  |  | **0.0124** |
| High | 1143 | 14.6 | 98.7 | 15 | 13.9 | 1.3 |  |
| Fair | 3738 | 47.6 | 99.0 | 37 | 34.3 | 1.0 |  |
| Low | 1938 | 24.7 | 98.3 | 33 | 30.6 | 1.7 |  |
| Very low | 1031 | 13.1 | 97.8 | 23 | 21.3 | 2.2 |  |
|  |  |  |  |  |  |  |  |
| **Expected future personal fulfillment at the social level** |  |  |  |  |  |  | **<.0001** |
| High | 3365 | 42.9 | 98.5 | 52 | 48.1 | 1.5 |  |
| Fair | 3502 | 44.6 | 99.2 | 27 | 25.0 | 0.8 |  |
| Low | 697 | 8.9 | 97.6 | 17 | 15.7 | 2.4 |  |
| Very low | 287 | 3.7 | 96.0 | 12 | 11.1 | 4.0 |  |
|  |  |  |  |  |  |  |  |
| **Expected future personal fulfillment at the educational/work level** |  |  |  |  |  |  | **<.0001** |
| High | 3928 | 50.0 | 98.8 | 46 | 42.6 | 1.2 |  |
| Fair | 2997 | 38.2 | 99.1 | 26 | 24.1 | 0.9 |  |
| Low | 612 | 7.8 | 97.1 | 18 | 16.7 | 2.9 |  |
| Very low | 314 | 4.0 | 94.6 | 18 | 16.7 | 5.4 |  |
|  |  |  |  |  |  |  |  |

**Supplementary Table 3.** Distribution of current and expected personal fulfillment in education and work among 7,959 high school students aged 15-17 according to the type of high school attended. Pavia, Lombardy region, Italy (2018-2022).

|  | **Lyceum** | | | **Technical High School** | | | **Vocational High School** | | |  |
| --- | --- | --- | --- | --- | --- | --- | --- | --- | --- | --- |
|  | **n** | **Column %** | **Row %** | **n** | **Column %** | **Row %** | **n** | **Column %** | **Row %** | ***p*-value (Chi-square test)** |
|  |  |  |  |  |  |  |  |  |  |  |
| **Present personal fulfillment at the educational level** |  |  |  |  |  |  |  |  |  | **0.0135** |
| High | 560 | 14.8 | 48.4 | 344 | 13.9 | 29.7 | 254 | 14.8 | 21.9 |  |
| Fair | 1851 | 49.0 | 49.0 | 1135 | 46.0 | 30.1 | 789 | 46.1 | 20.9 |  |
| Low | 922 | 24.4 | 46.8 | 626 | 25.4 | 31.8 | 423 | 24.7 | 21.5 |  |
| Very low | 447 | 11.8 | 42.4 | 361 | 14.6 | 34.3 | 246 | 14.4 | 23.3 |  |
|  |  |  |  |  |  |  |  |  |  |  |
| **Expected future personal fulfillment at the educational/work level** |  |  |  |  |  |  |  |  |  | **<.0001** |
| High | 2010 | 53.2 | 50.6 | 1196 | 48.5 | 30.1 | 768 | 44.9 | 19.3 |  |
| Fair | 1372 | 36.3 | 45.4 | 972 | 39.4 | 32.2 | 679 | 39.7 | 22.5 |  |
| Low | 269 | 7.1 | 42.7 | 187 | 7.6 | 29.7 | 174 | 10.2 | 27.6 |  |
| Very low | 130 | 3.4 | 39.2 | 111 | 4.5 | 33.4 | 91 | 5.3 | 27.4 |  |
|  |  |  |  |  |  |  |  |  |  |  |

**Supplementary Table 4**. Parameters’ estimates and multicollinearity statistics (i.e. Tolerance and VIF value).

|  | **Parameters estimates** | | | | | | |
| --- | --- | --- | --- | --- | --- | --- | --- |
| **Covariate** | **Degree of Freedom** | **Estimates** | **Standard error** | **t value** | **Prob > \|t\|** | **Tolerance** | **Variance Inflation Factor (VIF)** |
| Intercept | 1 | 0.98079 | 0.02581 | 38.00 | <.0001 | . | 0 |
| Sex | 1 | -0.01924 | 0.00264 | -7.28 | <.0001 | 0.96812 | 1.03293 |
| Type of school attended | 1 | 0.00533 | 0.00168 | 3.17 | 0.0015 | 0.94955 | 1.05313 |
| Age | 1 | 0.00293 | 0.00155 | 1.89 | 0.0594 | 0.99443 | 1.00560 |
| Nationality | 1 | 0.00247 | 0.00251 | 0.98 | 0.3264 | 0.97927 | 1.02117 |
| Survey year | 1 | 0.00200 | 0.00262 | 0.77 | 0.4437 | 0.99756 | 1.00245 |

**Supplementary Table 5**. Collinearity diagnostics.

| **Collinearity diagnostics** | | | | | | | | | |
| --- | --- | --- | --- | --- | --- | --- | --- | --- | --- |
| **N.** | **Eigenvalue** | **Condition Index** | **Proportion of variation** | | | | | | |
|  |  |  | **Intercept** | **Sex** | **Type of school attended** | **Age** | **Nationality** | **Survey year** |  |
| **1** | 5.56737 | 1.00000 | 0.00007953 | 0.00264 | 0.00454 | 0.00008355 | 0.00445 | 0.00266 |  |
| **2** | 0.16585 | 5.79390 | 0.00017613 | 0.11050 | 0.68532 | 0.00017137 | 0.01020 | 0.03205 |  |
| **3** | 0.13683 | 6.37867 | 0.00021327 | 0.00806 | 0.09268 | 0.00022310 | 0.92177 | 0.04978 |  |
| **4** | 0.09178 | 7.78832 | 0.00000268 | 0.41603 | 0.04213 | 0.00000546 | 0.01491 | 0.52441 |  |
| **5** | 0.03686 | 12.28950 | 0.01944 | 0.45908 | 0.17526 | 0.02241 | 0.04831 | 0.38439 |  |
| **6** | 0.00131 | 65.18769 | 0.98009 | 0.00368 | 0.00007969 | 0.97711 | 0.00035733 | 0.00671 |  |

N.: number

Both the VIF values (close to 1 for all the covariates) and collinearity diagnostics indicated the absence of multicollinearity among covariates.
